# A microsystem for in vivo wireless monitoring of plastic biliary stents using magnetoelastic sensors

**DOI:** 10.1038/s41378-024-00772-8

**Published:** 2024-10-31

**Authors:** Ramprasad M. Nambisan, Scott R. Green, Richard S. Kwon, Grace H. Elta, Yogesh B. Gianchandani

**Affiliations:** 1 EECS Department and Center for Wireless Integrated Microsystems and Sensing, University of Michigan, Ann Arbor, MI 48109 USA; 2 Division of Gastroenterology, Department of Internal Medicine, University of Michigan Health System, Ann Arbor, MI 48109 USA

**Keywords:** Electrical and electronic engineering, Biosensors

## Abstract

With an interest in monitoring the patency of stents that are used to treat strictures in the bile duct, this paper reports the investigation of a wireless sensing system to interrogate a microsensor integrated into the stent. The microsensor is comprised of a 28-μm-thick magnetoelastic foil with 8.25-mm length and 1-mm width. Magnetic biasing is provided by permanent magnets attached to the foil. These elements are incorporated into a customized 3D polymeric package. The system electromagnetically excites the magnetoelastic resonant sensor and measures the resulting signal. Through shifts in resonant frequency and quality factor, the sensor is intended to provide an early indication of sludge accumulation in the stent. This work focuses on challenges associated with sensor miniaturization and placement, wireless range, drive signal feedthrough, and clinical use. A swine specimen in vivo experiment is described. Following endoscopic implantation of the sensor enabled plastic stent into the bile duct, at a range of approximately 17 cm, the signal-to-noise ratio of ~10^6^ was observed with an interrogation time of 336 s. These are the first reported signals from a passive wireless magnetoelastic sensor implanted in a live animal.

## Introduction

The bile duct carries bile from the liver and gallbladder to the duodenum (small intestine). Strictures or narrowing of the bile ducts can occur in the setting of benign disease (such as chronic pancreatitis) or malignancy (such as pancreatic cancer)^1,2^. Bile duct obstructions have clinical consequences that range from elevated liver enzymes and jaundice to infections (cholangitis) and multiorgan failure. Endoscopic placement of plastic biliary stents (via a procedure called ERCP, or endoscopic retrograde cholangiopancreatography) is a mainstay therapy for relieving biliary strictures. Over a short but widely variable timeframe, plastic stents become occluded with accumulated “sludge” at a high rate (30–75% within 12 months)^3–6^. The major risk of biliary stent occlusion is bacterial infection of the bile ducts, or cholangitis, which can lead to life-threatening sepsis or widespread infection with high morbidity and mortality rates. Indeed, 20–40% of biliary stent patients present with occlusion and infection before their next planned intervention^7,8^. These emergency occlusions require hospitalization, antibiotics, and repeat ERCP to replace the stents (most often urgently or emergently).

In current medical practice, stent occlusion is suspected by elevated blood tests (i.e., liver enzymes) or the presence of biliary dilation on non-invasive imaging^9–14^. These are indirect indicators of occlusion. Elevations in liver enzymes that indicate stent obstruction are lagging indicators, manifesting after the stent is almost completely occluded and the patient is already at risk for infection. Imaging by ultrasound or magnetic resonance detects upstream biliary dilation and the lack of air in the bile duct (pneumobilia) which are indirect and imprecise signs of possible stent obstruction^15–22^. Neither indicator is based on an assessment of stent patency. Additionally, neither test is always performed at regular intervals and is often utilized only as the patient develops symptoms (for instance, recurrent pain or jaundice).

At the onset of the occlusion process, a protein layer starts to form on the stent surfaces^23,24^. After adhering strongly to this protein layer, bacteria form an extracellular matrix and biofilm. Altogether the matrix, biofilm, bacteria, and in some cases food particles in combination with bile form the sludge which occludes the stent. The progression of occlusion is associated with the accumulation of viscous mass on the interior of the stent.

Magnetoelastic materials are amorphous alloys that show magnetic and elastic properties. Such materials develop elastic strain in the presence of a magnetic field, and, as a result, generate their own magnetic field. This transduction mechanism has been widely used in anti-theft tags in the retail sector^25^ and has been widely investigated for wireless sensing applications. Typically, the magnetoelastic sensor is excited by an oscillating electromagnetic field, and the sensor response is detected by electromagnetic means as well, although acoustic and optical means of detection have also been used^26–33^. Optical means using a photoreceiver and laser are not feasible in an implanted situation as an implanted sensor is obscured from illumination by tissue. Acoustic means provide limited wireless range^34^. In this work, the electromagnetic domain is used for both exciting the sensor and detecting the sensor response with the help of inductive coils.

The concept of monitoring viscous mass accumulation within a lumen by using a wireless magnetoelastic sensor integrated with the stent has been reported previously by our team^35,36^. Ribbon-shaped structures made from metal alloys were used to form resonant sensors that were incorporated into a stent and interrogated wirelessly by electromagnetic waves in a benchtop set-up. The drop in resonant frequency and quality factor was correlated to mass accumulated on the sensor, representing progressive occlusion of the stent by bacterial sludge^35^. This sensing principle is discussed in detail in the Supplementary Note 1. This approach for monitoring the occlusion of a stent can enable decisions related to the nature and timing of the path of treatment, and avoidance of infections that may lead to costly emergent procedures or hospitalizations.

**Fig. 1:**
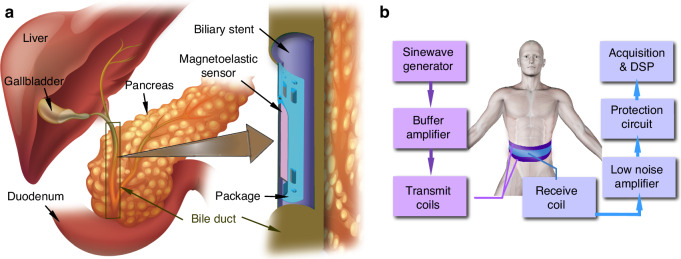
The biliary stent monitoring system. **a** Schematic of the magnetoelastic sensor integrated with the biliary stent for detecting occlusion. **b** Schematic of the interrogation module used for wireless communication with the sensor

The schematic diagram of the microsystem developed in this work is shown in Fig. 1. Figure 1a shows the bile duct and the implanted biliary stent, within which the packaged sensor attached is located. The microsystem consists of the magnetoelastic sensor and the package. The magnetoelastic sensor is comprised of a rectangular foil (8.25 mm length, 1 mm width, 28 μm thickness) on which two permanent magnets (1-mm length, 1-mm width, 60-μm thickness) are attached to provide the bias. The sensor is then packaged with a 3D printed polymer structure. Details of the sensor and package are in the Material and Methods section.

The interrogation subsystem (Fig. 1b) incorporates control electronics hardware as well as software for signal processing with a user interface. The electronics hardware includes several elements on the transmit side, including a sinewave signal generator, an amplifier, and the transmit coils. On the receive side it includes a receive coil, a low noise amplifier, an overvoltage protection circuit, and finally the data acquisition and digital signal processing equipment.

The focus of this work is to address challenges associated with the miniaturization of the sensor and its placement within the stent such that it accommodates ERCP and its tools. The size and location of the sensor, and the fluidic environment around it diminish the signal strength and present challenges for the interrogation subsystem. The interrogation subsystem must provide minimal feedthrough of the drive signal to the receive coil and must also provide a useful wireless range. Additionally, it must allow for variations in anatomy and positioning without compromising patient comfort. To reduce signal feedthrough, time domain decoupling is used in this work. In this method, the sensor is briefly excited, following which the ringdown response of the sensor is measured while the drive signal transmission is suspended. However, this ringdown response has low amplitude and a quickly decaying signal envelope. As such, the interrogation subsystem must have sufficient sensitivity and transmit-to-receive switching speeds to capture this signal. With regard to range, the magnetic field generated by the sensor decays with the distance, which limits the maximum distance between the sensor and the receive coil. Additionally, the magnetic field generated by the sensor decreases as the sensor size is scaled down dimensionally. A series of careful hardware choices and digital signal steps are used in this work in order to minimize the noise and achieve a high signal-to-noise ratio (SNR). Boosting wireless range enables clear signal detection over a wide range of anatomical variations.

The interrogation subsystem must be designed with consideration for clinical utility. The hardware must be user-friendly and introduce no discomfort to the subject or patient. The effective interrogation range should be long enough to reduce or eliminate the need for precise sensor-to-coil alignment; likewise, the recorded frequency response should be unaffected by sensor-to-coil alignment. Also, the interrogation time must be short enough to allow for subject comfort.

An additional advance presented in this work is the suppression of the increase in resonant frequency that would normally result from the miniaturization of the sensor; the increase in resonant frequency is avoided by increasing the structural mass of the sensor – attaching micromachined magnets that also provide the biasing magnetic field, thereby meeting two needs.

Another advance presented in this work is the incorporation of a sensor package residing within the stent in order to protect the sensor during ERCP and stent deployment. During the ERCP procedure, the stent and sensor are passed into the bile duct over an introducer – a tube that acts as a guide for the stent – and a guidewire. In the absence of a package, the introducer can cause damage to the sensor^29^. However, the package must also accommodate sharp longitudinal curvature imposed upon it during ERCP as the stent is deployed through an elevator in the instrument channel of the endoscope. Following its exit from the instrument channel, the stent must restore the sensor to its natural shape within the bile duct. This work uses a 3D printed polymeric package for flexibility and resiliency. This is presented in the Materials and Methods section.

The Materials and Methods section of this paper also describes the sensor, package, and interrogation subsystem in detail. The results section presents the in vivo experimental results with the system, and the discussion section follows afterward.

## Results

The animal test protocol was approved by the University of Michigan IACUC (protocol #6901). The ERCP procedure was performed by an expert interventional endoscopist on a 25 kg female domestic swine under general anesthesia. An endoscope commonly used in clinical practice (Olympus TJF Type Q180V side-viewing duodenoscope) was advanced through the stomach into the duodenum to the biliary orifice. Using standard endoscopic techniques, the bile duct was cannulated using a guidewire and catheter. A cholangiogram was obtained to delineate the anatomy before the stent was deployed over the guidewire using a standard stent deployment system (Oasis system, Cook endoscopy, Winston Salem, NC).

**Fig. 2:**
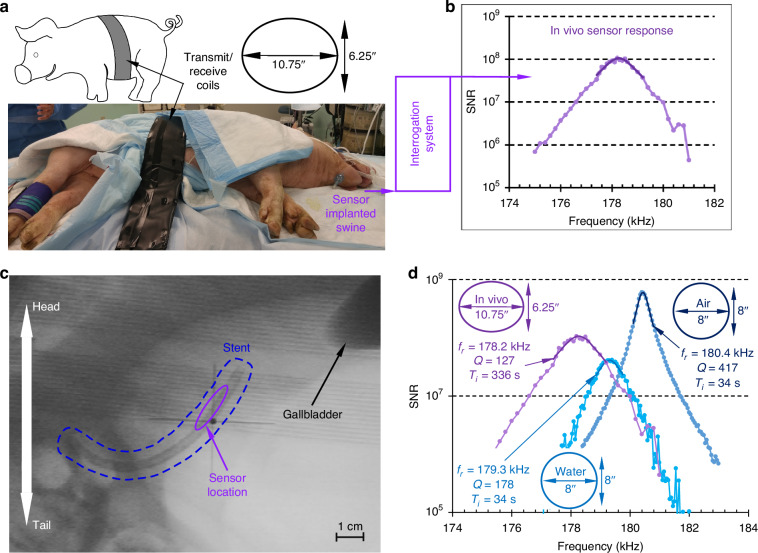
In vivo experiment. **a** Interrogation in progress, and the coils are placed like a belt on the swine specimen. **b** The first reported signals from a magnetoelastic sensor implanted in a swine specimen. **c** Fluoroscopic image of the stent after implantation in the swine. **d** Sensor response in vivo compared to the response on the benchtop in air and water

Next, for the interrogation, the transmit and receive belt coils were wrapped around the swine specimen, as shown in Fig. 2a. The in vivo measurements obtained from the sensor are shown in Fig. 2b. The SNR of this measurement was ~10^8^. These results represent the first successful wireless interrogation of a magnetoelastic sensor implanted in a live specimen. The fluoroscopic image taken after implantation is shown in Fig. 2c.

In Fig. 2d, in vivo measurements are compared to data obtained from benchtop tests in which the sensor embedded stents were immersed in water. The quality factor decreased from 178 (in water) to 127 (in vivo), and the resonant frequency shifted from 179.3 kHz (in water) to 178.2 kHz (in vivo). The coil dimensions used for the in vivo experiments were slightly different from benchtop experiments because of the swine anatomy. The interrogation time (T_i_) for benchtop tests was 34 s, while that for the in vivo tests was 336 s. The lower quality factor and resonant frequency observed in vivo are attributable to the sensor being immersed in bile rather than water; bile is more viscous than water. These results are indicative of the sensitivity of the sensor within the medium in which the sensor is immersed.

**Fig. 3:**
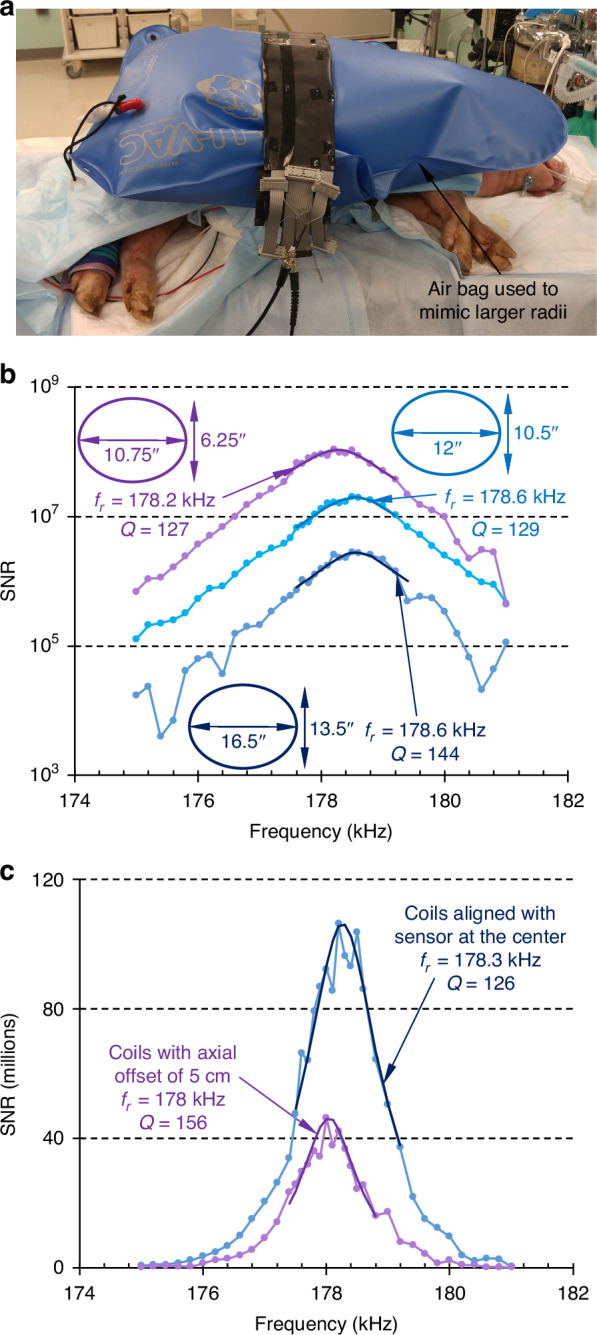
Extended in vivo experiments. **a** An inflated bag issued to mimic the increased waistline of a larger patient. **b** Sensor response for increased coil diameters mimicking patients with a larger abdomen, interrogation time 336 s. **c** Sensor response in vivo with misaligned coils and with an axial offset ~5 cm, interrogation time 340 s

In additional in vivo experiments, the interrogation coil size was increased to mimic the waistline of a larger specimen. The coil size was increased by inserting an inflated airbag between the subject and the coil as shown in Fig. 3a. The measurement obtained in these cases is also shown in Fig. 3b. Increasing the coil dimensions from major and minor elliptical diameters of 10.75” × 6.25” (27.3 cm × 15.9 cm) to 12” × 10.5” (30.5 cm × 26.7 cm) decreased the SNR to 25% of the previously measured value; however, the resonant frequency stayed within 0.4 kHz, and the quality factor within 2. A further increase in coil dimension to 16.5” × 13.5” (41.9 cm × 34.3 cm) resulted in a decrease of SNR to 15% of that measured with the initial coil dimensions; the shift in resonant frequency was only 0.4 kHz with a change in the quality factor of only 15. The interrogation time was 336 s.

Next, the coils were placed at an axial offset of 5 cm with respect to the sensor position to mimic a practical clinical situation. The sensor response in Fig. 3c shows that although the quality factor changed by 30, the resonant frequency only changed by 0.3 kHz (0.17%), and the SNR was ~4 × 10^7^. The interrogation time was 340 s. Overall, these results underscored the consistency of the measured values of resonant frequency despite some changes to the orientation and size of the coils.

Prior to the in vivo experiments, the microsystem was characterized on the benchtop for the placement and orientation of the sensor relative to the transmit/receive coils. In a set of experiments, the orientation of the sensor axis was varied with respect to the axis of the coils along which the applied magnetic field was at its maximum. It was found that the signal strength reduced to 12% with a 45^o^ misalignment in the orientation. With a 67.5^o^ incline the signal was reduced to 1%. In another set of experiments, the position of the sensor was also varied with respect to the center of the transmit/receive coil. The misplacement of the sensor along the axis of the coil at 7.6 cm from the center of the coil resulted in signal strength dropping to 1.5%. Details results from these experiments are explained in Supplementary Note 4. This characterization indicates the importance of aligning the belt coils around the subject during interrogation to obtain the best signal strength.

## Discussion

This investigation resulted in a system configuration that could wirelessly interrogate, in vivo and for the first time, a sensor enabled biliary stent implanted in a swine specimen. The signal feedthrough of the transmit signal was mitigated by time domain decoupling, and the wireless range was enhanced by the careful design of hardware and the use of digital signal processing. With an interrogation time of 336 s, the system provided a wireless range well exceeding 17 cm, and an SNR of ~10^6^. In tests that mimicked anatomical variations requiring the use of larger coil diameters, and potential coil misalignment, the resonant frequencies measured by the system changed upto only 0.17%.

Prior to the successful in vivo tests reported above, several attempts were unsuccessful because of shortcomings in the sensor and package, and in one case because the stent was not correctly sized for the swine specimen. Several critical requirements were recognized during these failed attempts, such as the need for a robust package to protect the sensor during deployment, and the need for a sophisticated interrogation subsystem for successful signal detection. These lessons learned were used in developing the sensor, package, and interrogation methods reported in this work.

The results provide guidance for several threads of future work. Because the sensor provided high SNR at a relatively large wireless range, there is potential for further miniaturization of the sensor. Several miniaturized sensors, each with a different resonant frequency, can be distributed across critical locations along the stent; this will allow for the localized detection of sludge accumulation. The nominal resonant frequencies of the distributed sensors can be controlled by varying the structural mass loading on each sensor. A sensor array could cover the entire inner lumen of the stent. Because an array of sensors will require an array of packages with the current approach, another prospect is to circumvent the need for a package by modifying the stent itself. A plastic stent that has a network of sensors incorporated within its wall could offer greater simplicity and scalability.

With regard to the interrogation subsystem, the current version uses multiple pieces of research-grade hardware. Ideally, an inexpensive, unified, and portable implementation is preferred, in order to facilitate more animal trials and ultimately human trials. The new module should be manufacturable at cost and volume scales that are commensurate with the intended use.

Further, the technology discussed in this paper can be extended to applications in other types of plastic stents including urethral stents and arterial stents. The low profile, passive, and wireless nature of the sensors supported by this interrogation approach may allow the replacement of invasive diagnostic methods. With a proper selection of sensor materials and designs, this technology can be even extended to use in metal stents.

The successful demonstration of the system described in this work suggests a pathway to inexpensive, real-time, outpatient procedures for directly monitoring stent occlusion, potentially aiding physicians in performing timely interventions and eliminating unnecessary invasive procedures in the course of post-implant care of stent patients. Ultimately, this will result in lower costs, fewer complications, and better outcomes. Further, regardless of the specific stent application, a wireless monitoring system with the sensitivity and spatial resolution targeted by this concept can provide clinical researchers with a map of the progression of stent occlusion. This could result in an improvement in the efficiency of important clinical studies, and the depth of knowledge gained by them. Continuous and long-term monitoring of the implanted sensor could provide more information on occlusion development as well as the health effects of the implanted module on a live animal.

## Materials and methods

### Sensor and package

The magnetoelastic sensor (8.25 mm × 1 mm footprint) was fabricated by bonding one 28 µm thick Metglas 2826MB structure with two 60 µm thick Arnokrome-5 mass loads. The Metglas structure was of ribbon shape, acting as a free-free beam with longitudinal mode resonant frequency of 215 kHz in air. Attaching the Arnokrome-5 structural mass load decreased the resonant frequency to 180 kHz. The bond was performed using Au/In transient liquid phase alloy. The mass loads were magnetized to provide a bias value of 3-4 G.

The sensor was then coated on both sides with aluminum oxide and then Parylene-C polymer (C_16_H_14_Cl_2_) to prevent corrosion and ensure biocompatibility. The aluminum oxide coating was 100-nm thick and provided by atomic layer deposition (ALD); the Parylene-C coating was 2-µm-thick parylene and provided by vapor phase deposition.

A 3D-printed polymer package (M3 crystal resin, 3D Systems, Rock Hill, SC), was used for protection and for integrating the sensor into the stent. The package was designed to reduce the deformation stress during deployment, keeping the maximum stress below the threshold for permanent deformation of the material. All of the materials used were known to be biocompatible, chemically inert, and electromagnetically transparent. The polymer package, with the sensor within it, was sewn into the wall of the stent. The cross-section of the sensor and package, together with wall of the sensor, occupied approximately 8 mm^2^ of the cross-sectional area of the open lumen, which is typically >130 mm^2^ in the target demographic of patients. Recognizing that stents are used to treat substantial obstructions of the lumen, even with the sensor package in place the open area of the lumen is greatly improved by the stent.

**Fig. 4:**
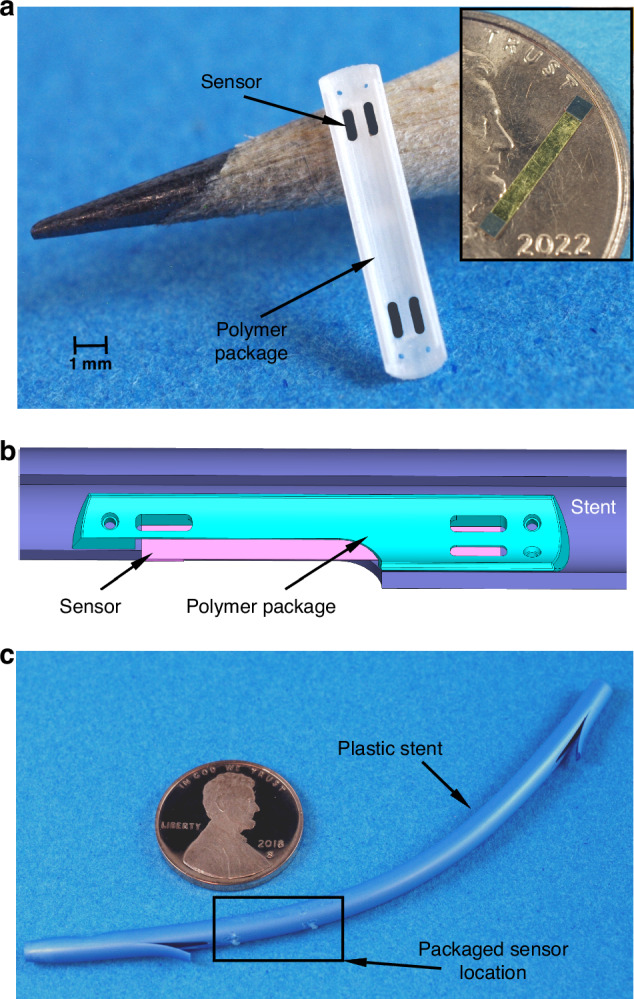
Custom sensor packaging and stent integration. **a** Magnetoelastic sensor in a package, inset shows the standalone sensor. **b** Schematic of the packaged sensor integrated onto the inner wall of the stent. **c** Stent integrated with the packaged sensor ready for implantation

A photograph of the sensor in the package is shown in Fig. 4a. The inset in Fig. 4a shows the standalone sensor on a penny. This packaged sensor was attached to the inner wall of the stent as illustrated in the schematic in Fig. 4b. Figure 4c shows the complete stent incorporating the packaged sensor, ready for ERCP.

### Interrogation subsystem – hardware

As noted previously, the interrogation subsystem (Fig. 1b) consisted of transmit coils for exciting the sensor magnetically and a receive coil for sensing the magnetic field produced by the vibrating sensor. The waveform generator (NI-5412, National Instruments, Austin, TX) along with the buffer amplifier (BUF634P, Texas Instruments, Dallas, TX) delivered the required sinusoidal voltage across and current through the transmit coils. The time domain decoupling approach, which was used to minimize the signal feedthrough, separated the interrogation cycle into a transmit segment and a receive segment. The transmit segment provided a sinusoidal burst during which the receive circuit was blocked, whereas in the receive segment the transmission was suspended and the sensor ringdown was detected. This sensor-generated magnetic field created a current through the receive coil, which was amplified and converted to a voltage using a low noise amplifier (LNA) (SR-570, Stanford Research Systems, Sunnyvale, CA) and captured by the data acquisition equipment (NI-6115, National Instruments, Austin, TX) for further processing. A protection circuit was used to block and shunt large input currents away from the low noise amplifier; these currents would otherwise arrive at the low noise amplifier input during the transmit segments of the interrogation cycle because of mutual induction between the transmit and receive coils. The details of these elements are presented in Supplementary Note 2.

**Fig. 5:**
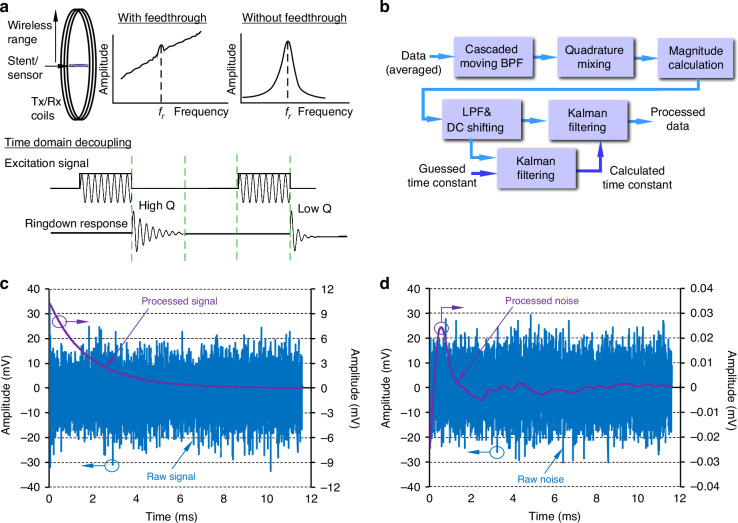
Signal integrity, conditioning, and processing. **a** Wireless range and feedthrough challenge associated with an interrogation module and time domain decoupling approach. **b** Schematic of digital signal processing techniques implemented in Labview^TM^. **c** Signal, and **d** noise before and after all digital signal processing steps

The signal after the LNA remained noisy and required further processing. A series of digital signal processing techniques were used to filter the noise and recover the sensor signal. These included using a cascaded band-pass filter (BPF) and quadrature mixing to filter in the targeted frequency range; envelope detection followed by a low-pass filter (LPF) to further suppress the noise; and a Kalman filter algorithm to detect the ring-down signal. Figure 5a illustrates the time domain decoupling used in this work. The flow diagram of the signal processing sequence is shown in Fig. 5b. Details of these techniques are explained in Supplementary Note 3. The raw signal and the signal after all the digital signal processing steps are shown in Fig. 5c. The processed signal is filtered out from the noise as shown in this figure. For comparison, Fig. 5d shows the raw signal and the processed signal for noise when there is no sensor signal evident after digital signal processing. Overall, the resulting improvement in SNR allowed the wireless range to be increased (up to 33 cm) to accommodate a wide range of anatomical variations.

### Interrogation subsystem – transmit/receive coils

Considering clinical utility, rigid cylindrical coils are less accommodating of anatomical variations, so the coils were reimagined in the style of a belt. The belt consisted of a transmit coil and a receive coil. Since the sensors used in this work vibrate longitudinally, the transmit coil was used to generate the alternating magnetic field in the preferred orientation. The magnetic field generated by the sensor was similar in shape to that of a bar magnet, but of an oscillating nature. A single receive coil was used to receive the field generated by the sensor response. The transmit and receive coils were fabricated from standard ribbon cables, with standard snap-together cable connectors that were offset by a single wire, allowing the formation of a continuous coil. All the coils were enclosed in nylon fabric for an added layer of structural integrity and electrical isolation. The position of the coils was maintained within the nylon fabric by stitching between each coil. Hook and loop connectors (Velcro^TM^) were used for the form-fitting placement of the coil module around the midsection of the torso while allowing the dimensions of the resulting inductive loop to be adjusted within a useful range. This belt was placed around the subject, such that the sensor-integrated stent was approximately in the middle of the coil, as shown in Fig. 1b, and attached by the aforementioned cable connectors.

The transmit and receive coils were designed to match their impedance with associated equipment. The transmit coils were driven by the buffer amplifier, and the magnetic field generated by the transmit coil was maximized by optimizing the number of turns of the coil to match the coil impedance to the specified output impedance of the buffer amplifier (50 Ω) and thus allowing the maximum current to be delivered to the coil. To estimate the coil impedance, the transmit coil can be modeled as a multi-turn coil with the inductance given by^37^:

1 $$L=\frac{{N}^{2}{{r}_{c}}^{2}}{25.4(8{r}_{c}+11a)}{mH}$$

where *r*_*c*_ (~0.1 m) is the radius, *a* is the width of the coil and *N* is the number of turns in the coil. This equation is valid for *2r*_*c*_ > *a* > *0.2r*_*c*_. For the wire used, the width per turn was 1.25 × 10^−3^ m, resulting in total width, *a*, of *N* × 1.25 × 10^−3^ m. The resistance of the coils was relatively small compared to the reactance presented by the inductance and thus could be neglected in the total impedance calculations. The effective coil impedance was then:

2 $${Z}_{{coil}}=\frac{{N}^{2}{{r}_{c}}^{2}\omega }{25.4(8{r}_{c}+0.00125N)}m\varOmega$$

where *ω* (=2π*f*, *f* = 155 kHz – the expected resonant frequency of the resonator) is the radial frequency of the signal. Equating the coil impedance to 50 Ω and using Eq. (2), it was found that two parallel coils with 14 turns maximized the generated magnetic field at the expected sensor frequency. A current of 0.125 A through each of these coils provided a magnetic field of ≈0.2 G.

The sensing side of the interrogation module consisted of the receive coil connected in series with the protection circuit, LNA, and data acquisition device. The equivalent resistance of these circuit elements was estimated to be 365 Ω. The number of turns of the receive coil was optimized for the maximum current generation at a given magnetic flux density, as the LNA is a transimpedance amplifier. The receive coil acted as a voltage source while the magnetic field generated by the magnetoelastic sensor coupled with it. The voltage *V* generated across the coil by varying magnetic field is given by Faraday’s law of electromagnetism:

3 $$V=-N\frac{d\Phi }{{dt}}=-N\omega \Phi$$

where $\varPhi$ is the magnetic field, *ω* is the frequency and *t* is the time. The current generated through the circuit is given by:

4 $${I}_{c}=\frac{V}{{Z}_{{coil}}+{Z}_{{Load}}}$$

where *Z*_*coil*_ is the impedance of the coil, and *Z*_*Load*_ is the impedance of the load (365 Ω). Since this was a multi-turn coil, we could again use Eq. (2) to estimate the coil impedance. Simplifying Eq. (5) shows that *Z*_*coil*_ is approximately proportional to the square of *N*. This gives:

5 $$\,{Z}_{{coil}}=m{N}^{2}\omega$$

where *m* is a constant of proportionality. Combining Eqs. (3), (4), and (5) gives the current through the coil as:

6 $${I}_{c}=\frac{N\omega \Phi }{m{N}^{2}\omega +{Z}_{{Load}}}$$

At a given frequency, the highest current magnitude is generated when *N* is chosen such that *Z*_*coil*_ is equal to *Z*_*Load*_. Using Eq. (2), it was found that a single coil with 28 turns would provide the highest sensitivity. Details are provided in Supplementary Note 5.

## Supplementary information

Supplementary Notes (Clean Version)
