# Supplementary material for: A microsystem for in vivo wireless monitoring of plastic biliary stents using magnetoelastic sensors
Source: Microsyst Nanoeng. 2024 Oct 31;10:159. doi: 10.1038/s41378-024-00772-8 (PMC11526028; doi:10.1038/s41378-024-00772-8)
Supplement: Supplementary file 1 — Supplementary Notes (Clean Version) [file 41378_2024_772_MOESM1_ESM.docx]

SUPPLEMENTARY NOTES

**A Microsystem for *In Vivo* Wireless Monitoring of Plastic Biliary Stents Using Magnetoelastic Sensors**

Ramprasad M. Nambisan, Scott R. Green, Richard S. Kwon,

Grace H. Elta, Yogesh B. Gianchandani

**Supplementary Note 1: Magnetoelastic Sensing for Mass Loading**

Magnetoelastic devices vibrate at their mechanical resonance in the presence of an oscillating magnetic field at that resonant frequency. The vibration is due to the strain caused by the alignment with an applied field of elongated magnetic domains. The mechanical vibration of the magnetoelastic device launches its magnetic waves, which can be sensed externally by using an inductive coil [R1]. This physical phenomenon follows the linearized constitutive relations coupling stress, strain, magnetic field, and magnetic flux density [R2]:

$\left[ \varepsilon\right]=\left[ s^{H} \right].\left[ \sigma\right]+\left[ d \right].\left[ H \right]$ (1)

$\left[ B \right]=\left[ d \right]^{T}\left[ \sigma\right]+\left[ \mu^{\sigma} \right].\left[ H \right]$ (2)

where [*ε*] is the strain tensor, [*σ*] is the stress tensor, [*s^H^*] is the compliance matrix at constant magnetic field intensity, [*d*] is the magnetostrictivity matrix, [*H*] is the magnetic field intensity, [*B*] is the magnetic flux density, [*µ^σ^*] is the permeability matrix at constant stress. These equations can be used in analytical modeling of the sensors or actuators.

As indicated in the Introduction section of the manuscript, the resonant frequency, and the quality factor of a magnetoelastic sensor varies depending on the mass accumulated on the sensor as illustrated in Fig. S1.


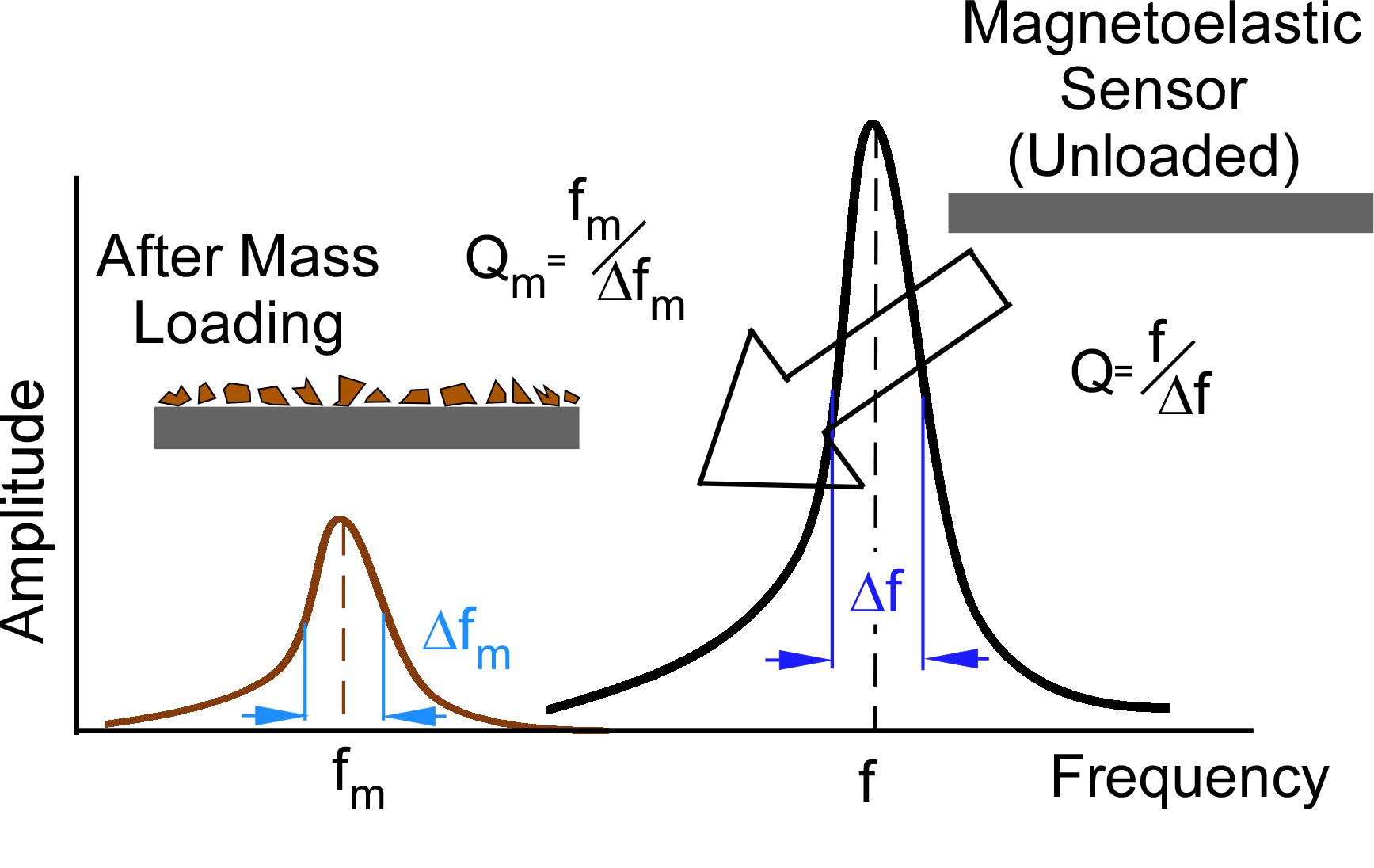


Fig. S1: Deposition of viscoelastic material onto the resonant sensor results in a shift in resonant frequency as well as (typically) a reduction of the quality factor.

The drop in resonant frequency and quality factor was correlated to mass accumulated on the sensor for a uniform mass loading in equations as [R3]:

$\frac{\Delta m}{M}=\left( \frac{f_{o}}{f} \right)^{2}-1$ (3)

$Q=\frac{\sqrt{\left( \frac{\pi}{2L} \right)^{2}k(M+\Delta m)}}{c}$ (4)

where *Δm* is mass accumulated, *M* is the mass of the resonator, *f_o_* is the resonant frequency of an unloaded sensor and *f* is the resonant frequency after mass accumulation, Q is the quality factor, L is the length of the resonator, k is the effective stiffness and c is the viscosity of the medium. By measuring the change in resonant frequency and quality factor, one can determine mass accumulation on the sensor.

**Supplementary Note 2: Hardware implementation**

As discussed in the main manuscript, in the subsection Interrogation Subsystem – hardware (Materials and Methods section), the hardware includes a waveform generator and buffer amplifier at the transmit side and an LNA, protection circuit and data acquisition device at the receive side. The details of the interrogation subsystem are illustrated in detail in Fig. S2.


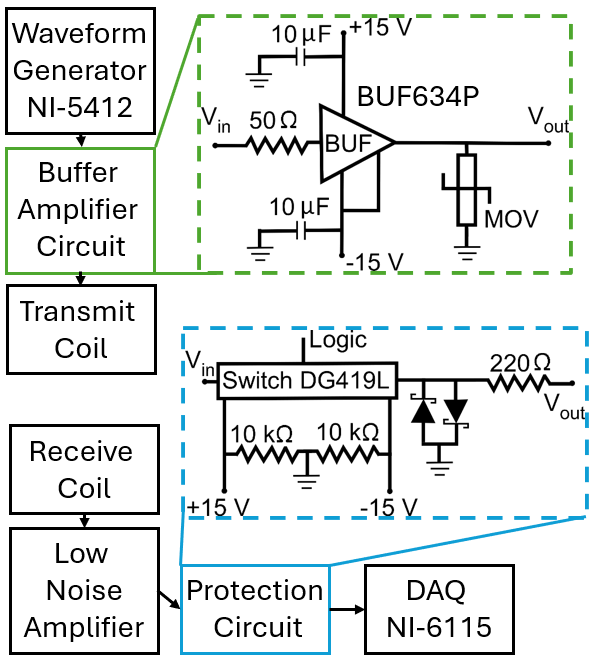


Fig. S2 | Interrogation subsystem in detail.

The hardware components on the sensor signal reception side are the LNA, protection circuit, and data acquisition device. The LNA amplifies the received signal while introducing minimal noise, which helps in improving the SNR, which will subsequently improve the wireless range. The protection circuit is for protecting the LNA from overdrive, especially during the transmit time. Finally, the received signal is collected to a PC using a data acquisition device. The selection of various settings on the LNA – including the passband of onboard filters, sensitivity, and high-bandwidth or low noise mode – determines its response. The passband for the LNA is set to 10 kHz – 300 kHz after estimating the sensor resonance frequency as 155 kHz. The sensitivity is optimized experimentally at 20 µA/V for the best signal to noise ratio while avoiding overload of the LNA input or output. The high-bandwidth mode is utilized to ensure the LNA can capture signals near 100 kHz. The noise specification for the LNA with these sensitivity and mode settings is $60 pA/\sqrt{\mathrm{Hz}}$.

The protection circuit, placed between the output of the receive coils and the input of the LNA, is used to avoid overloading of the LNA. The protection circuit consists of an analog CMOS switch and antiparallel Schottky diodes (Vishay BAT46-TR). The switch in the protection circuit is used to avoid overloading of the LNA input due to the mutual induction between the transmit coils and the receive coil during the transmit period. The switch logic is programmed in LabVIEW so that it is “off” during the transmit period and “on” during the receiving period. As a backup protection measure, Schottky diodes along with a series resistance are used for limiting the maximum current to the LNA even if the switch fails in a short condition. The maximum input that can be fed to the LNA is 5 mA peak. A current equal to 5 mA through the series resistance results in a voltage that turns on these Schottky diodes, providing an alternate path for the current and thus avoiding any damage to the LNA. A “blank” signal, turning off the gain of the LNA, is also used to avoid output overloading during the transmit period. This blank signal sets the gain of the amplifier at 0 dB during the transmit phase and then sets the gain back to the 20 µA/V level during the receiving phase.

Finally, the amplified signal from the LNA is collected to a PC using a data acquisition device for further digital signal processing and display of results. This device collects the voltage data from the LNA at a 1 MS/s sampling rate during the receive period. The signal processing steps are detailed in the next section.

**Supplementary Note 3: Digital Signal Processing**

The signal after the LNA is still noisy, and the extraction of any useful information requires further processing. A series of digital signal processing techniques are used to minimize the noise and recover the sensor signal. These techniques help to improve the SNR, and an improved SNR allows improvement of the wireless range. The flow diagram of these techniques is shown in Fig. 3b (Main manuscript).

The data acquired into the PC is divided into two; the first half represents the sensor signal, and the second half is considered to be the background noise. This helps to evaluate the signal to noise ratio (SNR) by taking a real-time noise signature into account. In the first step of signal processing, the strength of the white noise present in the signal is reduced by taking an average of several data traces of the signal. Because the jitter of the acquisition timing is small compared to the time periods of interest, this process does not degrade the signal information. The signal after averaging is illustrated in Fig. S3.


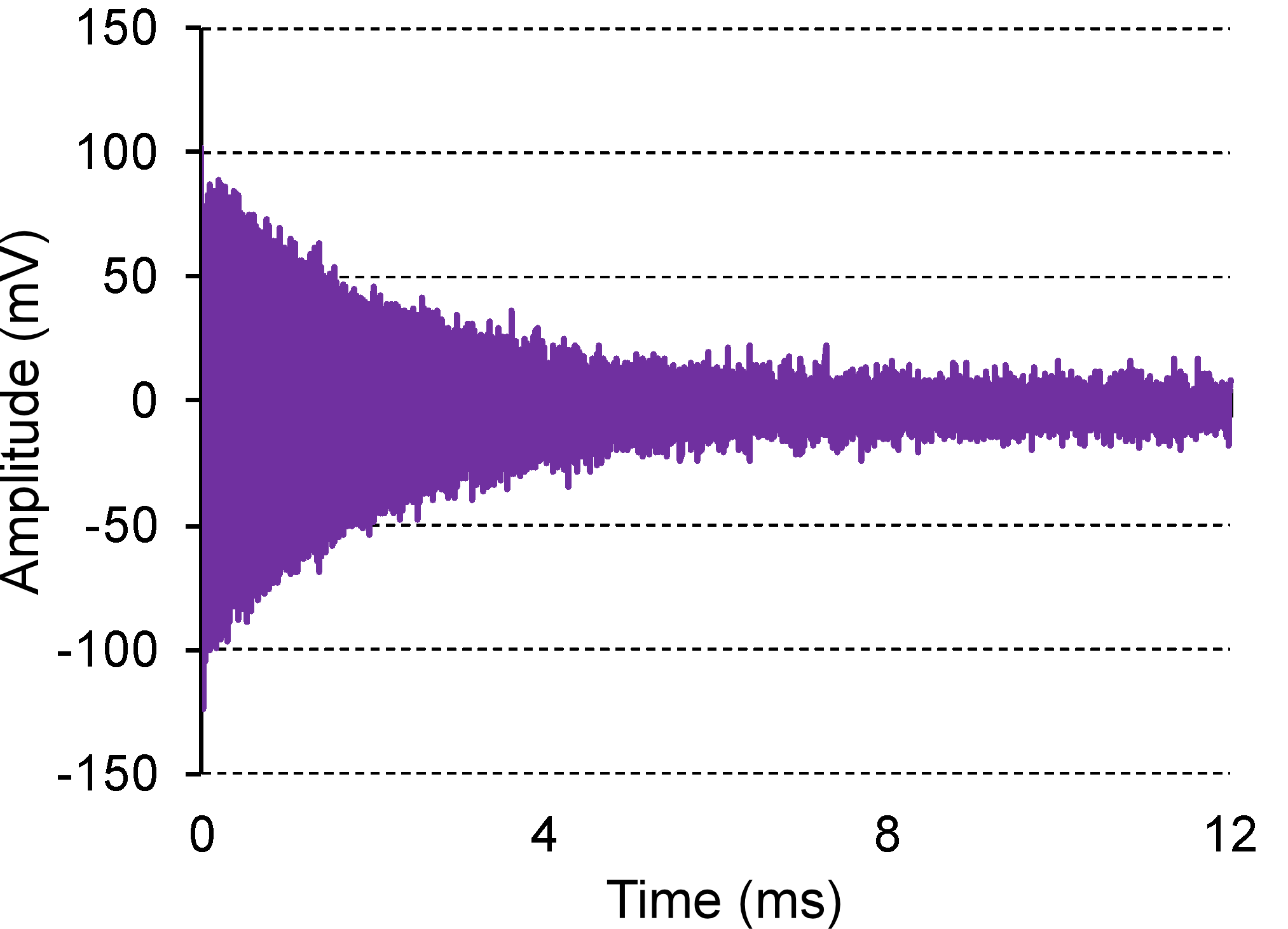

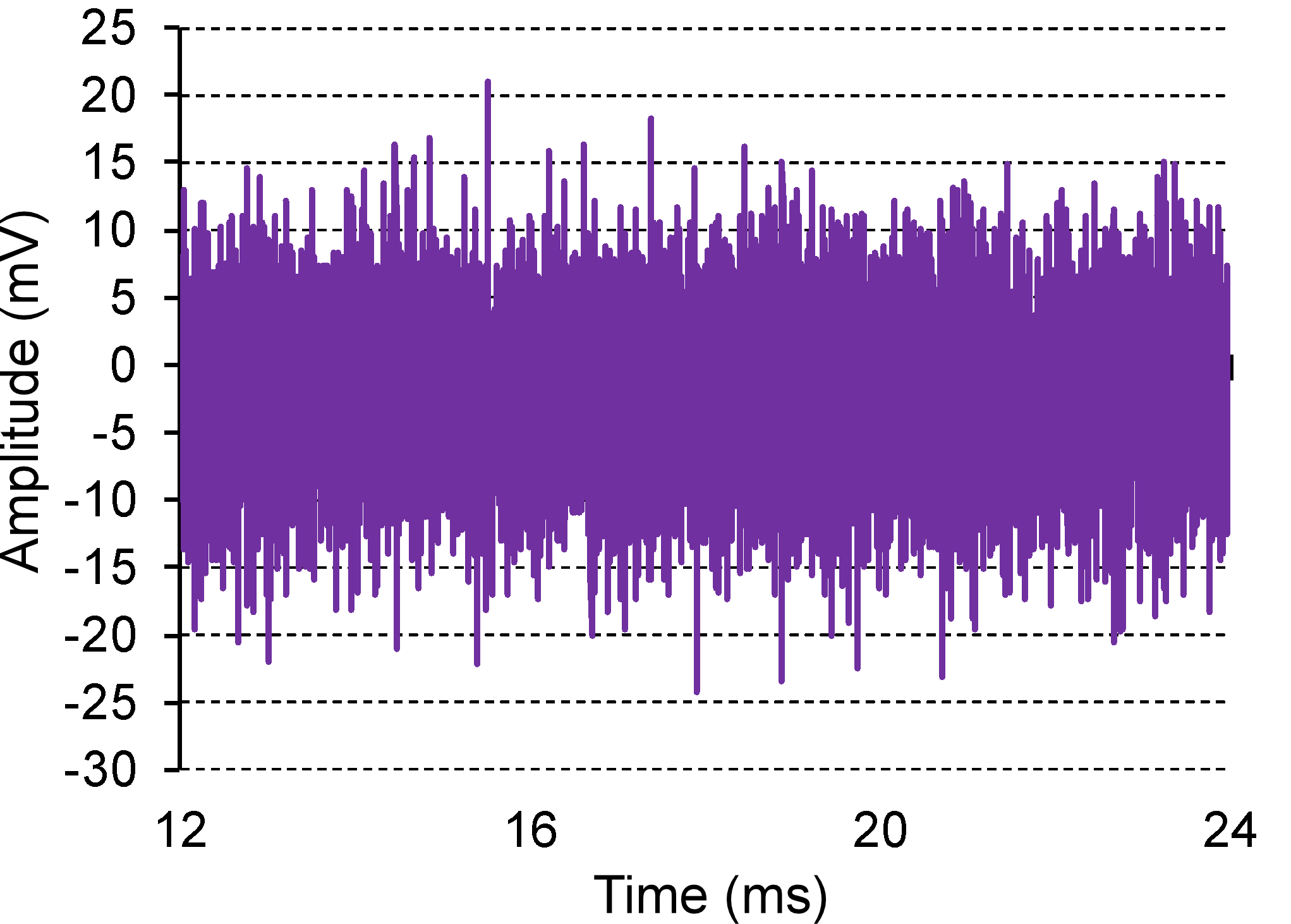


Fig. S3 | Left – Signal data after averaging. Right – Noise data after averaging.

The next step implemented in signal processing is a bandpass filter. The bandpass filter designed is a finite impulse response (FIR) filter. An FIR filter is selected over an Infinite Impulse Response (IIR) filter because of its guaranteed stability and an exactly linear phase [R4, R5]. This property of having linear phase results in constant group delay for all frequency components of the signal while filtering. This is an important advantage when compensating for group delay. A linear phase FIR filter is designed using the Remez exchange algorithm to minimize the maximum error between the target frequency response and the designed filter frequency response. This error specification yields filters with equi-ripple or Chebyshev error behavior [R6]. A cascaded filter approach is used here by using two bandpass filters with slightly offset bandpass frequencies in order to achieve a narrow filter with central frequency f_0_. This is illustrated in Fig. S4.


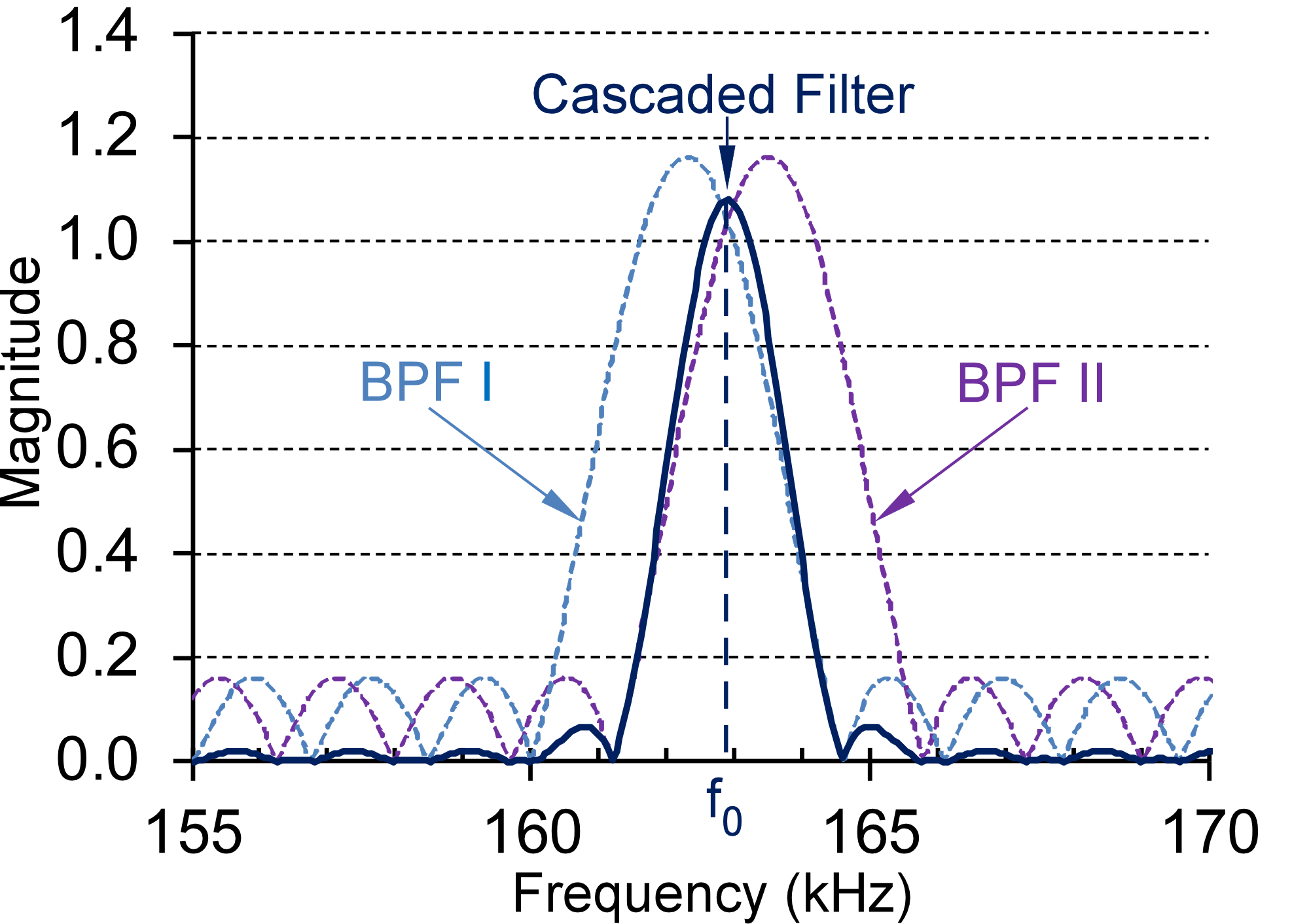


Fig. S4 | Cascaded moving band pass filter implementation.

These filters used in this step are also moving filters, in the sense that the filter specifications are varied with respect to excitation frequency in real-time. The central frequency (f_0_) of the effective filter will be the excitation frequency. The raw signal after cascaded bandpass filtering is illustrated in Fig. S5.


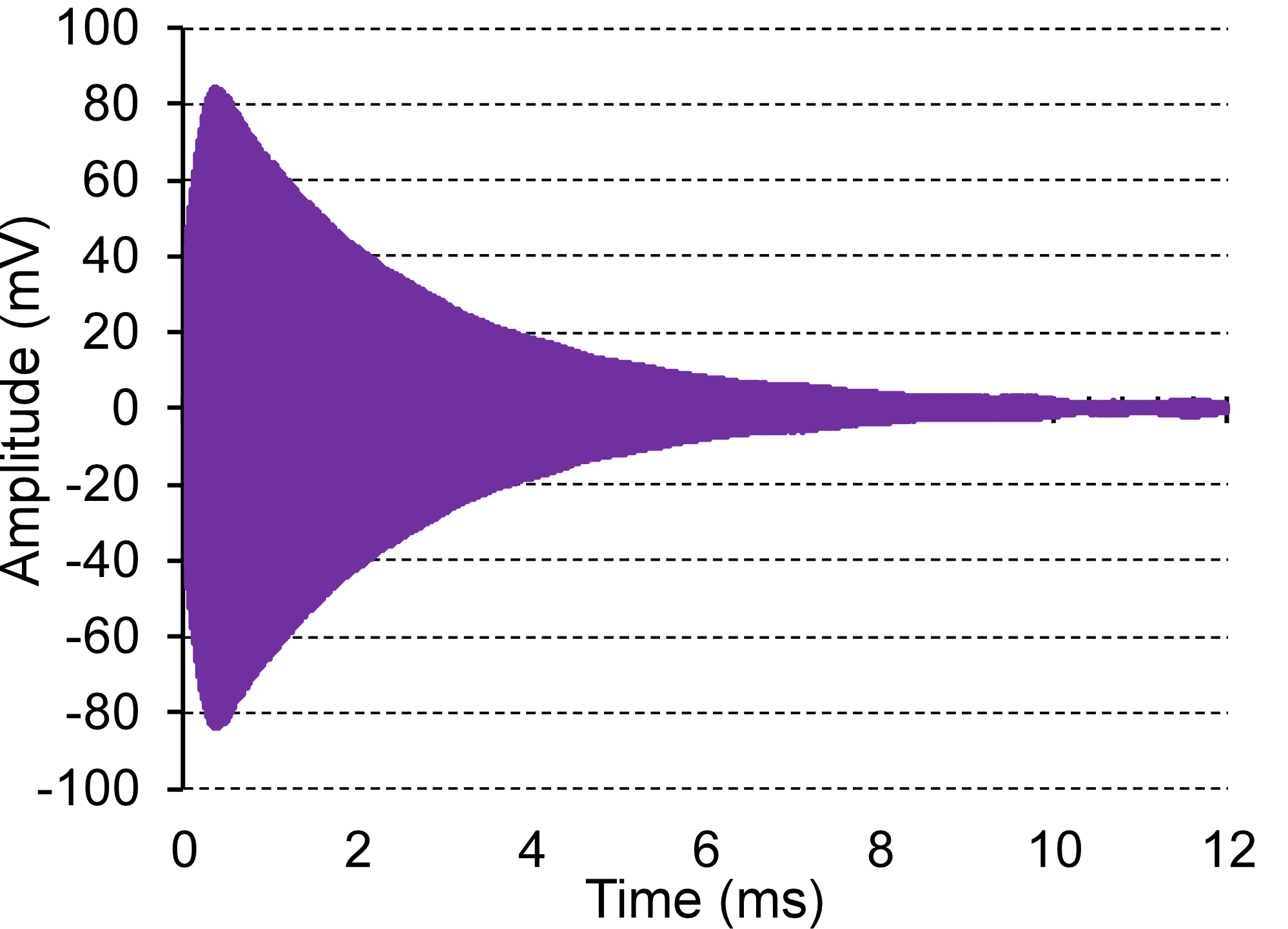

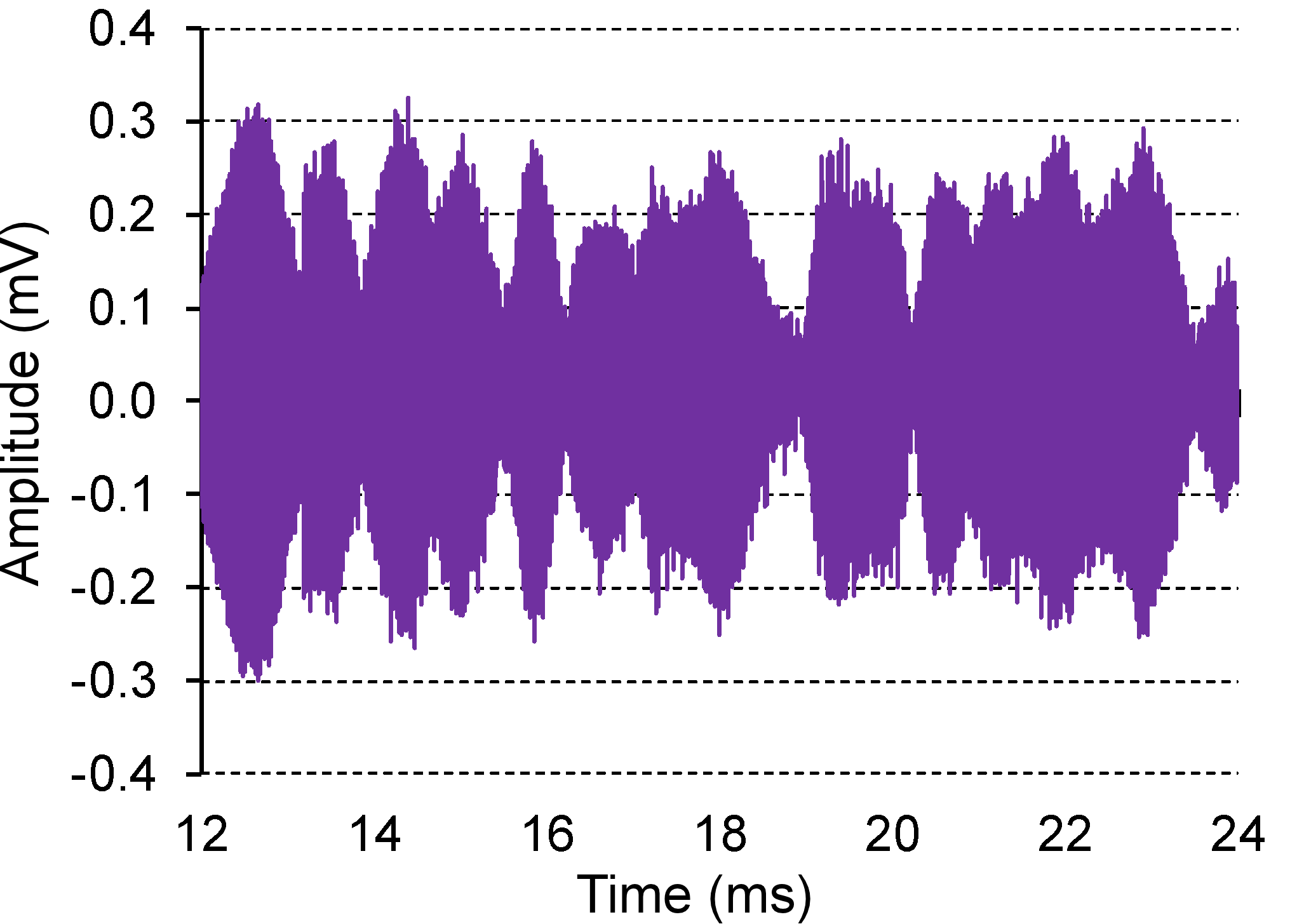


Fig. S5 | Left – Signal data after cascaded band pass filtering. Right – Noise data after after cascaded band pass filtering.

The filtered signal from the bandpass filter is then quadrature mixed by multiplying with two sinusoid signals in quadrature phase relation at the excitation frequency, and the magnitude of the mixed signals are calculated in order to isolate the frequency component of interest. High-frequency components are removed by using a low pass filter afterward, and the envelope of the signal amplitude is extracted. The low pass filter is an equi-ripple low pass FIR filter designed using the Remez exchange algorithm with a passband frequency of 1 kHz and stopband frequency of 3 kHz. A DC component is present in the observed measurements, and this is eliminated by subtracting the mean noise value from the observed noise. This brings the noise to randomly oscillate around zero instead of a constant value. The signal is also shifted with the same value to keep the measurements consistent. The raw signal after this step is illustrated in Fig. S6.


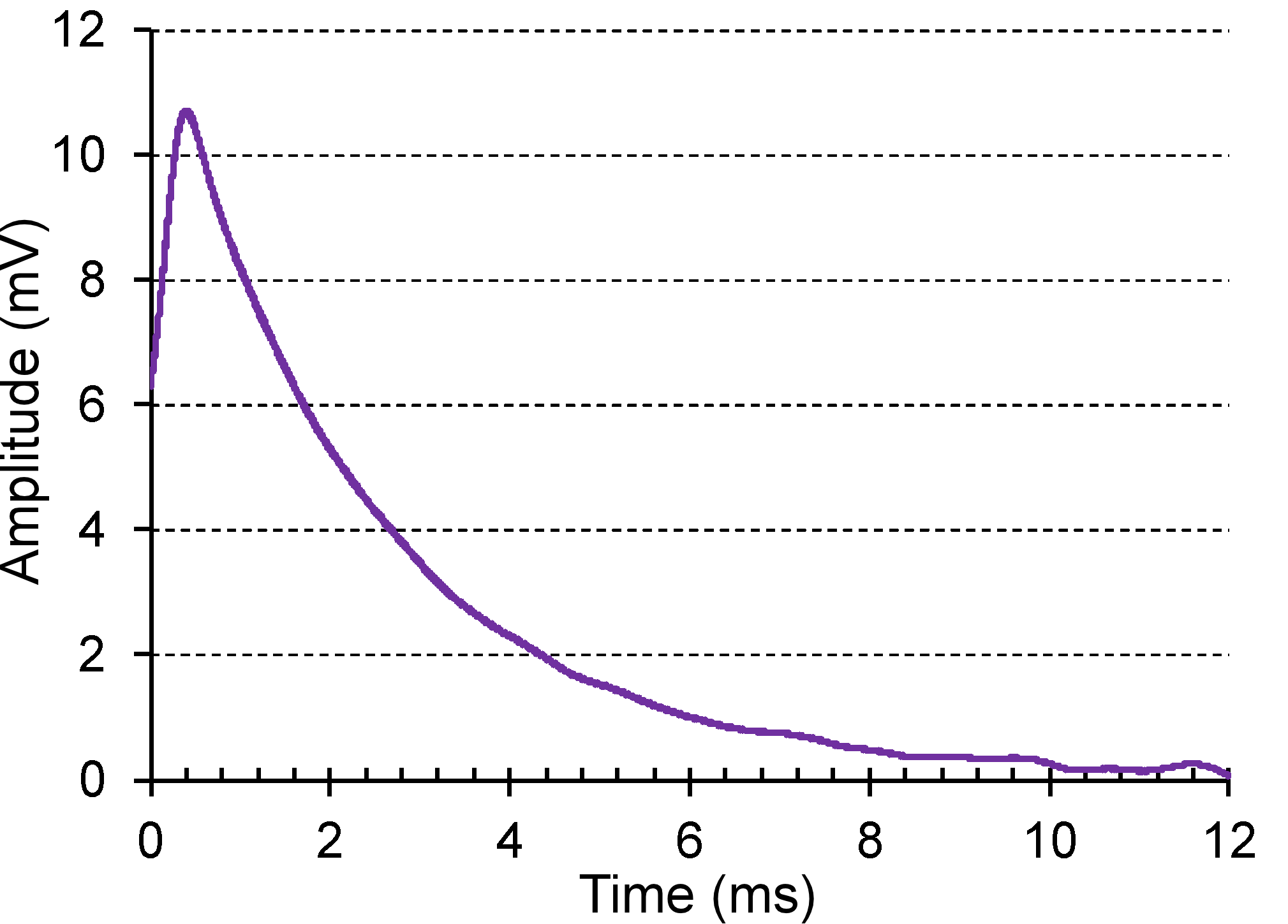

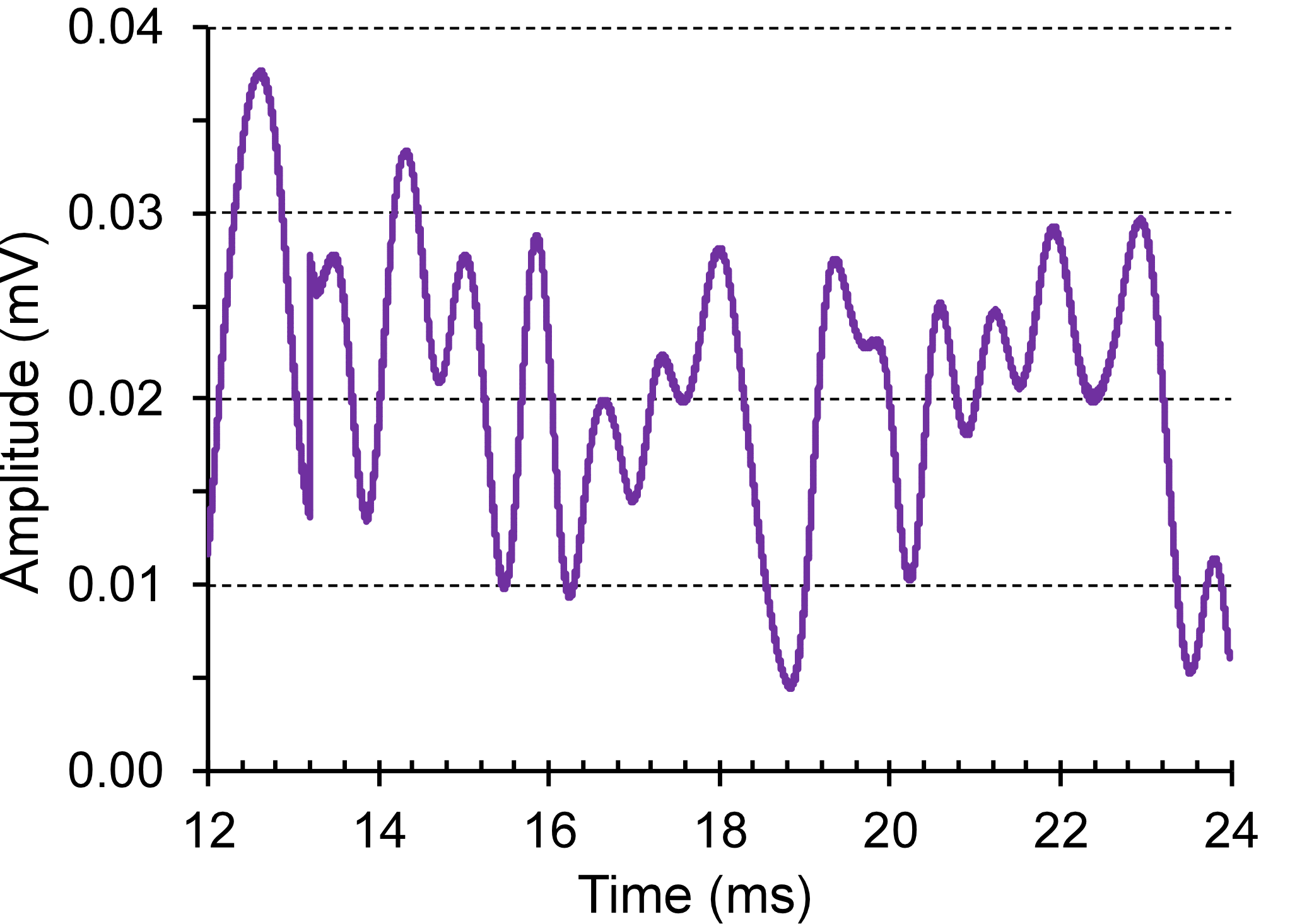


Fig. S6 | Left – Signal data after quadrature mix and low pass filter. Right – Noise data after quadrature mix and low pass filter.

Further, a Kalman filter (KF) is implemented exploiting the knowledge that the envelope of the response of a magnetoelastic sensor excited via a time domain decoupled signal is a first order exponentially decaying signal. Using a KF, we can estimate the state of a process (system) by using the observation, knowledge of the system behavior and noise, and a set of mathematical equations [R7-R9]. This technique helps to reduce the error in the measurements of the system by predicting the expected response. We have good knowledge of the expected response of our system, as well as an ability to characterize the noise at every frequency (by measuring over a longer period of time than during which the ringdown signal is substantial). Thus, KF is useful, allowing the noisy ringdown signal to be smoothed and noise to be suppressed substantially. The KF steps are repeated for all the data points to obtain the entire filtered ringdown signal/noise envelope. The signal and noise data after Kalman filtering is illustrated in Fig. S7.

S7. The raw signal and the signal after all the DSP steps are shown in Fig. 3b and Fig. 3c in the main manuscript.


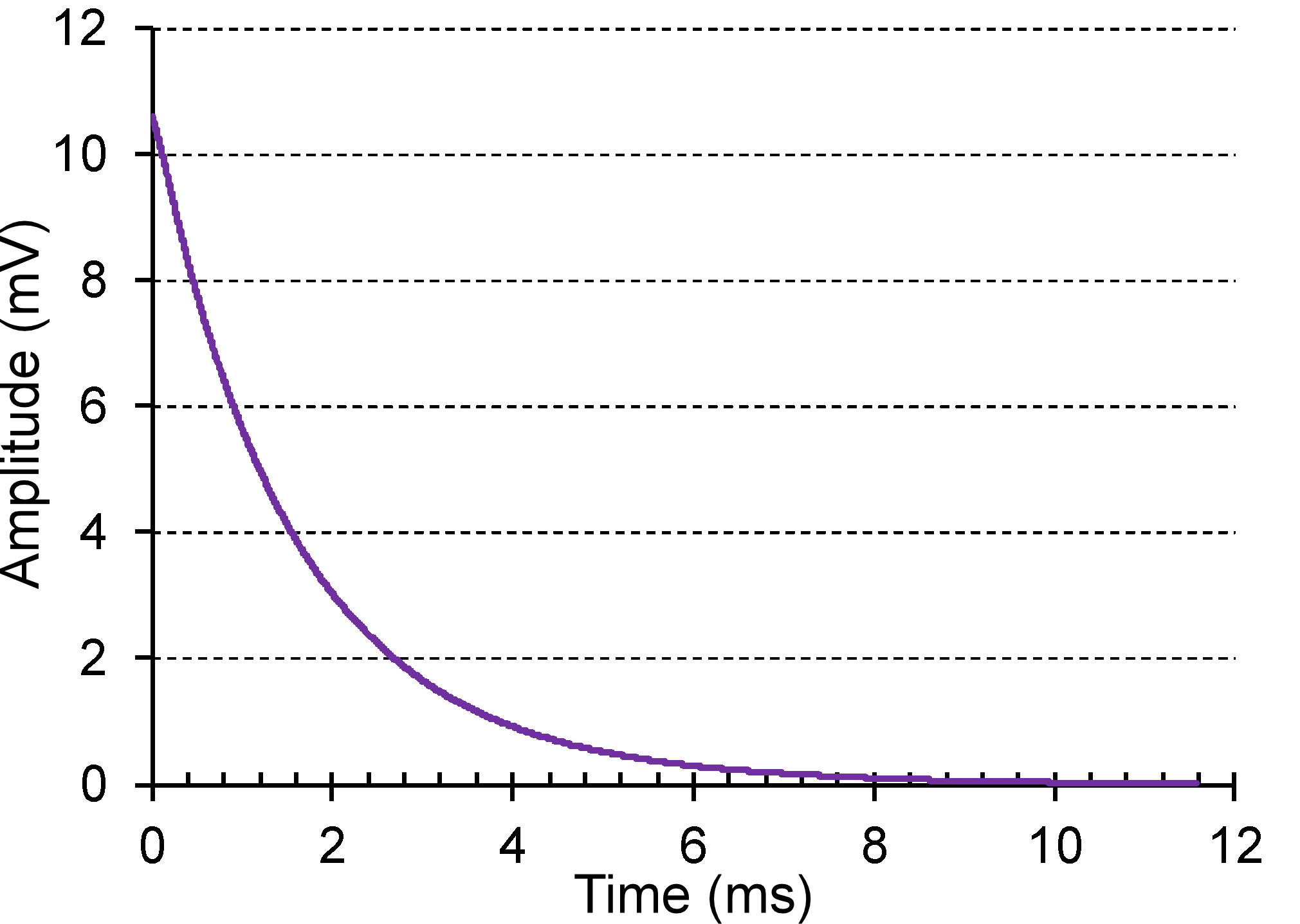

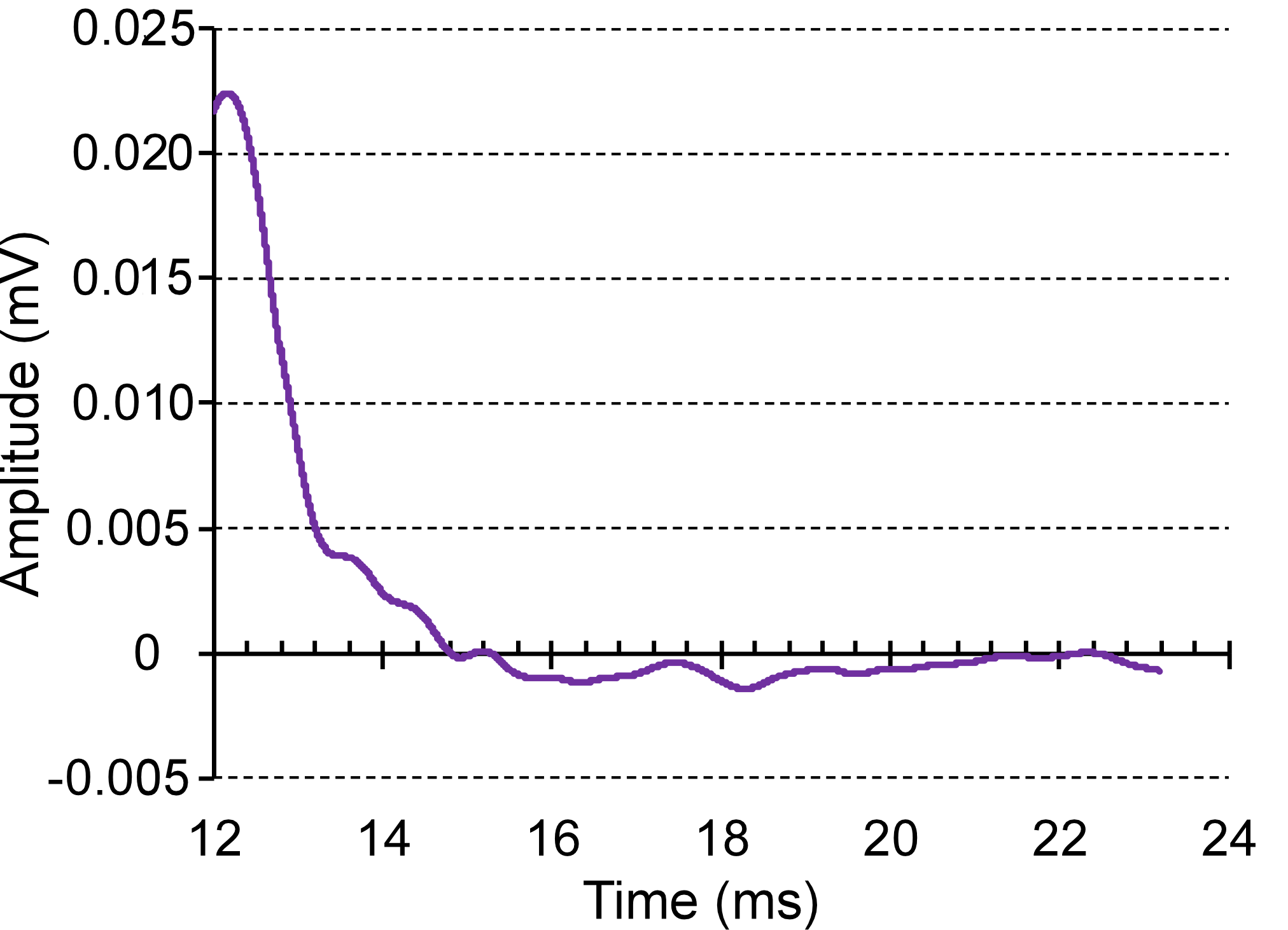


Fig. S7: Left – Signal data after Kalman filtering. Right – Noise data after Kalman filtering.

The received signal rings down with a characteristic time constant, and as such, the signal strength is predominant in initial cycles and well above the noise. To benefit from all the time that signal is above the noise, the summed squared amplitude (SSA) is calculated instead of just the maximum amplitude of the ring down signal;

$SSA=\sum_{t=0}^{n} \left| x_{t} \right|^{2}$ (7)

where *x_t_* = amplitude of the signal at sample *t* after DSP. The ratio of SSA calculated for the signal and real-time noise is defined as the SNR. The value of *n* is optimized by calculating the SSA for all values of *n* at the resonant frequency, and then utilizing the value of *n,* which results in the highest SNR.

Implementation of the cascaded moving BPF and LPF after quadrature mixture results in an inherent group delay. Since these filters are FIR, they have linear phase and hence constant group delay. For group delay compensation (GDC), group delay for each filter is calculated, and an equivalent number of samples were padded with zeroes at the beginning of the signal. This zero padding helps in minimizing the distortion in the signal due to group delay and compensate for this effect [R10].

**Supplementary Note 4: Benchtop Experiments**

The benchtop experiments were conducted to characterize and demonstrate the utility of the interrogation module for interrogating a magnetoelastic sensor. In a clinical situation, after the implantation of sensor integrated stent in a patient, the stent position and orientation can vary according to the patient-specific position and orientation of the bile duct; this cannot be visualized externally. As such, these benchtop experiments investigate the dependence of the signal strength on the angle between the longitudinal axis of the sensor and the AC excitation field and the dependence of the signal strength on the sensor position relative to the transmit/receive coil. For these experiments, the belt coil (transmit and receive coils) was wound around a 20 cm diameter cardboard barrel. The sensor, packaged in a stent, was placed at the center of the belt coil in the air.

For the first set of experiments, the signal dependence on the angle between the axis of the sensor and the AC excitation field was examined. The angle between the longitudinal axis and the exciting AC field was varied, and the change in the signal strength was observed, as in Fig. S8.


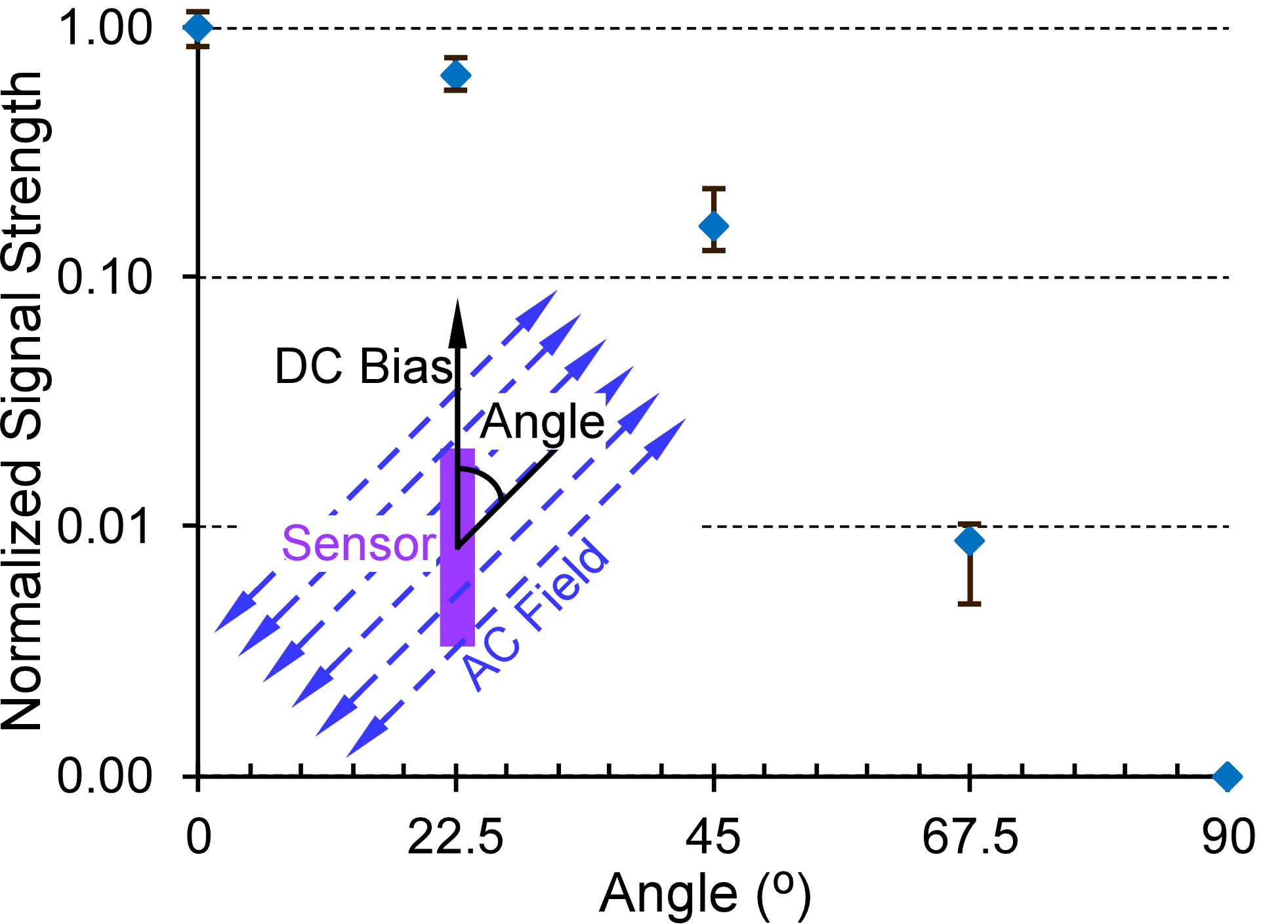


Fig. S8 | Change in signal strength with the angle of the sensor with respect to the axis of the coil.

The signal strength was reduced significantly to 12% for the case where the sensor was inclined 45^o^ with respect to the axis of the interrogation coils. For the 67.5^o^ inclined case, the signal was reduced to 1%. The decrease in the signal strength was close to an exponentially-decaying function of the angle between the sensor axis and the transmit/receive coil axis. These results indicate that the magnetic field generated by the sensor is mostly in the direction of the axis of the coil and hence, any deviation from it reduces the signal strength considerably. This indicates the importance of having the longitudinal axis of the sensor aligned with the axis of the belt coil.

In the next set of experiments, the position of the sensor was varied for several axial (z) and radial (*r*) distances from the center of the belt coil and the variation in signal strength was noted as shown in Fig. S9.


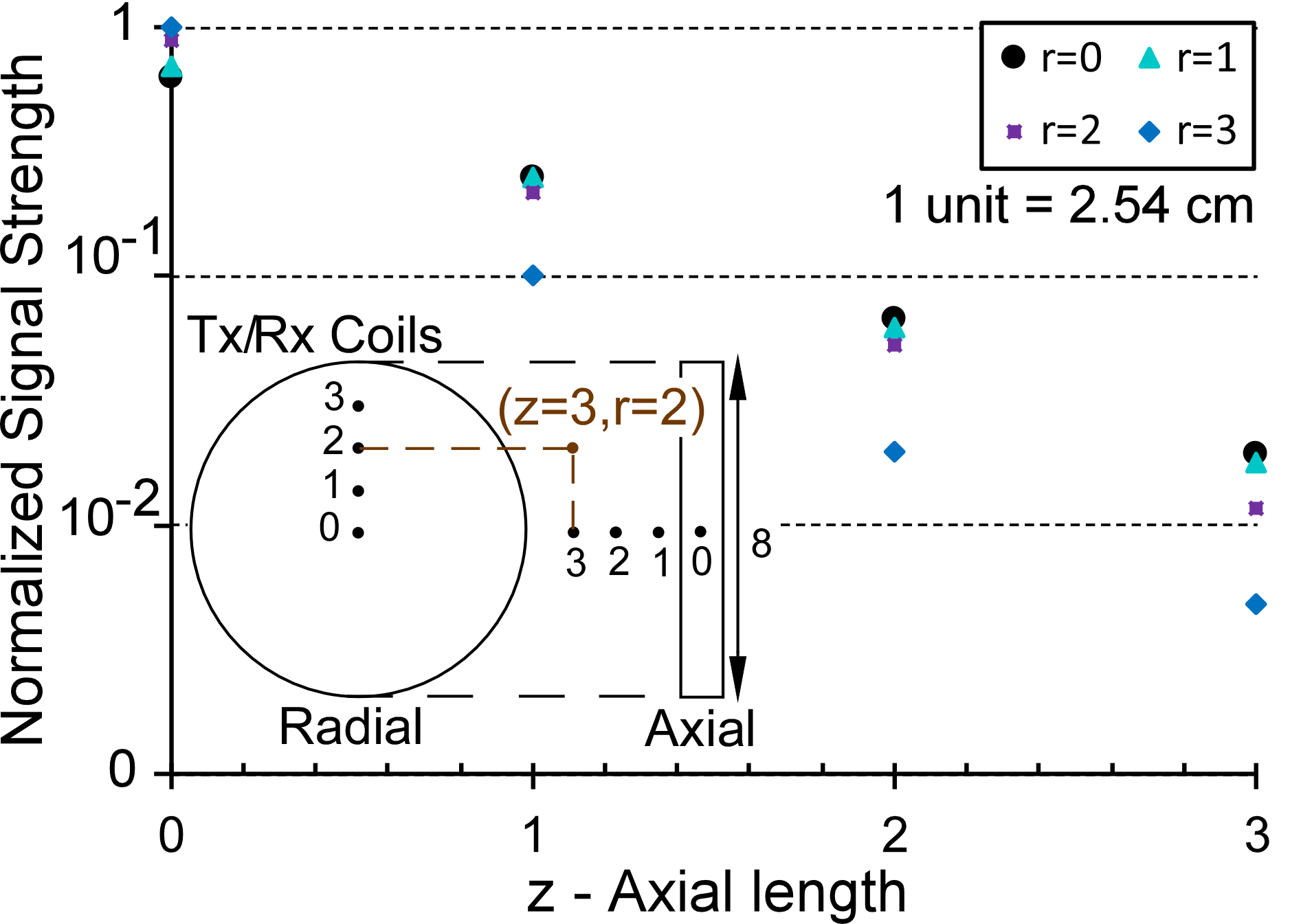


Fig. S9 | Change in signal strength with the position of the sensor with respect to transmit/receive coil. *r* and *z* represent the radial and axial distance, respectively, from the center of the coil (similar to the cylindrical coordinate system).

For this test, the longitudinal axis of the sensor and the axis of the barrel were parallel. For *r* =0 and *z* =7.62 cm, the signal dropped to 1.5% of the signal captured at *r* =0 and *z* =0. As the sensor position was moved away from the center of the coil along the axis of the coil, the signal strength reduced exponentially. At the same time, the sensor shows higher signal strength when it is closer radially to the transmit/receive coil for the case *z* = 0, but a lower signal nearer the coil when in other axial locations. Also, signal strength reduced to 4% when the coil diameter is increased from 20 cm to 30 cm. That is a 25-fold decrease in signal strength for a 1.5-fold increase in wireless range.

In summary, the sensitivity of the interrogation module depends on the characteristic pattern of the excitation field as well as the sensor field. More excitation results in more signal strength, but at the same time, the sensor signal needs to be coupled well with the receive coil to detect the signal. The best-case scenario here is to have the sensor close to the transmit/receive coils in the same plane of the coil, i.e., no axial distance separating the middle of the coils from the sensor location.

**Supplementary Note 5: Interrogation Subsystem Transmit/receive coil**

As discussed in the subsection Interrogation Subsystem – Transmit/receive Coil within the Materials and Methods section of the main manuscript, the transmit and receive coils were fabricated considering impedance matching and also the clinical utility. The size and dimension of the final coil used are illustrated in Fig. S10.


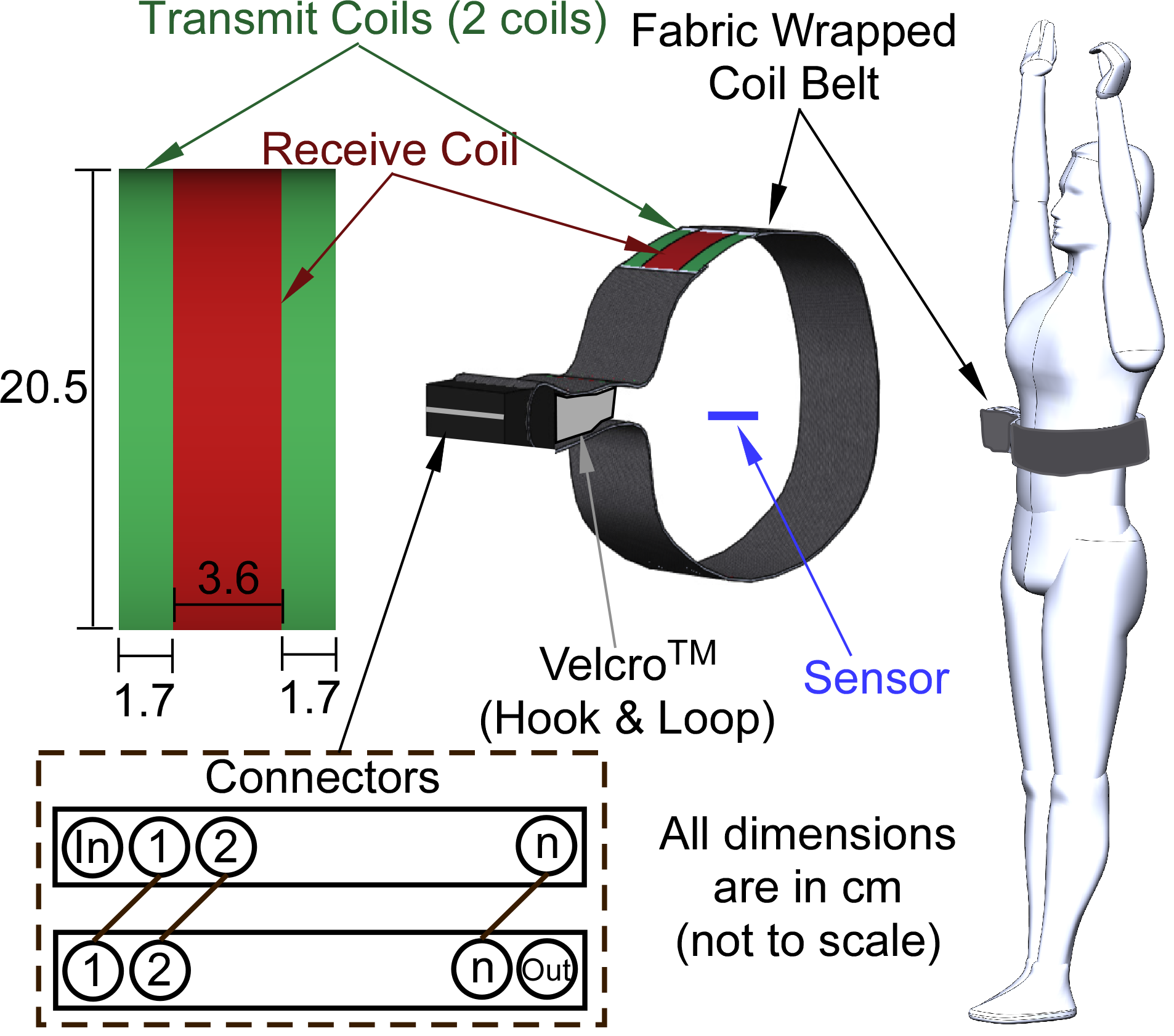


Fig. S10 | Coil structure and clinical utility. The offset connection for the fabrication of a continuous coil from ribbon cable is also illustrated in the lower left inset.

Both the transmit coil and receive coil were wrapped with a fabric in the style of a belt and will be wrapped around the subject for the interrogation as shown in the figure.

Supplementary References

[R1] C.A. Grimes, C.S. Mungle, K. Zeng, M.K. Jain, W.R. Dreschel, M. Paulose, and K.G. Ong, “Wireless magnetoelastic resonance sensors: A critical review,” *Sensors*, *2*(7), pp.294-313, 2002.

[R2] H. W. Katz, “Electrostrictive and Magnetostrictive Systems,” *Solid State Magnetic and Dielectric Devices*, New York: John Wiley and Sons, 1959

[R3] S. R. Green, “Wireless Magnetoelastic Monitoring of Biliary Stents,” Doctoral dissertation, University of Michigan, 2009.

[R4] L. Litwin, “FIR and IIR digital filters,” *IEEE potentials*, Vol. 19, No. 4, pp.28-31, 2000.

[R5] D. F. Elliott, “Handbook of digital signal processing: engineering applications,” *Academic press*, 2013.

[R6] A. E. Cetin, O. N. Gerek, Y. Yardimci, “Equiripple FIR filter design by the FFT algorithm,” *IEEE Signal Processing Magazine*, Vol. 14, No. 2, pp.60-64, 1997.

[R7] R. E. Kalman, “A new approach to linear filtering and prediction problems,” *Journal of Basic Engineering*, Vol. 82, No. 1, pp.35-45, 1960.

[R8] M. S. Grewal, “Kalman filtering,” *Springer Berlin Heidelberg*, pp.705-708, 2011.

[R9] C. K. Chui, G. Chen, “Kalman filtering with real time applications,” *Applied Optics*, Vol. 28, pp.1841, 1989.

[R10] J. Borkowski, J. Mroczka, “LIDFT method with classic data windows and zero padding in multifrequency signal analysis,” *Measurement*, Vol. 43, No. 10, pp.1595-1602, 2010.
